# Radical Scavenging Is Not Involved in Thymoquinone-Induced Cell Protection in Neural Oxidative Stress Models

**DOI:** 10.3390/antiox12040858

**Published:** 2023-04-01

**Authors:** Christopher Krewenka, Sandra Rizzi, Chi Huu Nguyen, Marcin Delijewski, Lars Gille, Katrin Staniek, Johanna Catharina Duvigneau, Khaled Radad, Andrea Müllebner, Barbara Kranner, Rudolf Moldzio

**Affiliations:** 1 Institute of Medical Biochemistry, Department of Biomedical Sciences, University of Veterinary Medicine, 1210 Vienna, Austria; 2 X4 Pharmaceuticals, 1030 Vienna, Austria; 3 Department of Pharmacology, Faculty of Medical Sciences in Zabrze, Medical University of Silesia, 40-055 Katowice, Poland; 4 Institute of Pharmacology and Toxicology, Department of Biomedical Sciences, University of Veterinary Medicine, 1210 Vienna, Austria; 5 Department of Pathology, Faculty of Veterinary Medicine, Assiut University, Assiut 71515, Egypt

**Keywords:** neuronal cell culture, primary mesencephalic cell culture, neuroblastoma cell line, thymoquinone, rotenone, MPP^+^, oxidative stress, superoxide radical scavenging

## Abstract

Thymoquinone (TQ), an active compound from *Nigella sativa* seeds, is often described as a pharmacologically relevant compound with antioxidative properties, while the synthesis of TQ in the plant via oxidations makes it inapplicable for scavenging radicals. Therefore, the present study was designed to reassess the radical scavenging properties of TQ and explore a potential mode of action. The effects of TQ were studied in models with mitochondrial impairment and oxidative stress induced by rotenone in N18TG2 neuroblastoma cells and rotenone/MPP^+^ in primary mesencephalic cells. Tyrosine hydroxylase staining revealed that TQ significantly protected dopaminergic neurons and preserved their morphology under oxidative stress conditions. Quantification of the formation of superoxide radicals via electron paramagnetic resonance showed an initial increase in the level of superoxide radicals in the cell by TQ. Measurements in both cell culture systems revealed that the mitochondrial membrane potential was tendentially lowered, while ATP production was mostly unaffected. Additionally, the total ROS levels were unaltered. In mesencephalic cell culture under oxidative stress conditions, caspase-3 activity was decreased when TQ was administered. On the contrary, TQ itself tremendously increased the caspase-3 activity in the neuroblastoma cell line. Evaluation of the glutathione level revealed an increased level of total glutathione in both cell culture systems. Therefore, the enhanced resistance against oxidative stress in primary cell culture might be a consequence of a lowered caspase-3 activity combined with an increased pool of reduced glutathione. The described anti-cancer ability of TQ might be a result of the pro-apoptotic condition in neuroblastoma cells. Our study provides evidence that TQ has no direct scavenging effect on superoxide radicals.

## 1. Introduction

*Nigella sativa* seeds, as well as its main pharmacologically active compound, thymoquinone (TQ) (2-isopropyl-5-methyl-1,4-benzoquinone), have been widely used in traditional medicine for several thousand years [1]. While *Nigella sativa* seeds were used in the past as a natural treatment to support health and cure illnesses, modern studies demonstrate that TQ has anti-bacterial, anti-inflammatory, and anti-cancer activities [2,3,4,5,6]. The promising pharmacological potential of TQ is still part of scientific curiosity, which is demonstrated by the increasing number of publications related to new and primarily unexpected pharmacological activity. Recently, several publications about TQ and its effects against COVID-19 have been published, which reflect the high expectations placed on TQ [7,8]. Besides the use of TQ throughout the centuries, it has been also reported that TQ may be an effective compound in neurodegenerative disease models [9]. Based on a study by Badary et al., the protective properties of TQ have been attributed partially to its ability to scavenge superoxide radicals ex vivo [10].

Neurodegenerative disorders are often caused or accompanied by oxidative stress [11]. In Parkinson’s disease (PD), for instance, oxidative stress is considered to be one of the main reasons for the decline of the dopaminergic neurons, besides the aggregation of α-synuclein and the induction of inflammation [12]. Likewise, in Alzheimer’s disease (AD), cell degeneration seems to be related to inflammation and oxidative stress [13,14]. The neuroprotective effects of TQ are often attributed to its antioxidative effects. For example, Kandeil and his colleagues postulated that the beneficial effect of TQ against cisplatin-induced neurotoxicity is due to its antioxidant activity [15]. Kennedy et al. observed in human SH SY5Y neuroblastoma cells, a cellular model for AD, that TQ showed a lowered amyloid β oxidation and a decreased nitric oxide level as a result of the antioxidative capacity of TQ [16]. Additionally, in an animal model for PD, Ebrahimi and his colleagues attributed the beneficial effects of TQ to its antioxidant effects [17]. Thus, TQ might be a promising candidate to protect neurons attacked by radicals.

Nevertheless, whether the radical scavenging or secondary antioxidative effects of TQ are responsible for the neuroprotection, is still unclear. Although, the radical scavenging hypothesis proposed by Badary et al. and Kruk et al. is widely recognised, the antioxidative or superoxide radical scavenging properties of TQ cannot clearly be deduced from its chemical structure [10,18]. In detail, the proposed biosynthesis of TQ in plants is a hydroxylation of ρ-cymene to carvacrol catalysed by an oxidoreductase. Carvacrol is further oxidised to thymohydroquinone (THQ), which is finally oxidised to thymoquinone [19]. Thus, that radical scavenging properties of TQ are not as obvious as the inclusion of TQ or its reduced form, THQ, into cellular antioxidative mechanisms.

Therefore, the first aim of our study was to investigate the potential of TQ to counteract oxidative stress. In our in vitro PD model, neuroblastoma, and a mesencephalic primary cell culture were treated with mitochondrial complex I inhibitors, rotenone and 1-methyl-4-phenylpyridinium (MPP^+^), leading to the increased formation of superoxide radicals. The second aim was to reassess the radical scavenging properties of TQ and to explore its potential mechanism of action. Hence, electron paramagnetic resonance (EPR), a method to quantify the formation of superoxide radicals, was applied to a neuroblastoma cell culture treated with TQ.

## 2. Materials and Methods

Pregnant OF1/SPF mice were purchased from the Institute for Laboratory Zoology and Veterinary Genetics (Himberg, Austria. Cell culture studies are animal replacement studies. In Austria, the preparation of primary cultures does not require authorization but does involve a notification requirement to the governmental authorities. The animals were kept and handled in accordance with the guidelines in the European Union Directive (2010/63/EU) on the use of animals for scientific purposes.

### 2.1. Preparation of Dissociated Mesencephalic Cell Cultures

Primary mesencephalic cell cultures were prepared according to the protocol of Gille et al., with minor modifications [20]. In brief, pregnant mice were anesthetized using CO_2_ and sacrificed by cervical dislocation. The embryos were carefully isolated. The ventral mesencephala were excised, the meninges were removed, and the tissues were mechanically cut into small pieces. After trypsin treatment, the tissues were triturated and dissociated in a serum-containing medium (Dulbecco’s Modified Eagle Medium, high glucose (DMEM (with 4500 mg/L glucose and sodium bicarbonate, phenol red, without L-glutamine, and sodium pyruvate)) supplemented with 8.7 mM HEPES, 9.7 mM glucose, 1.7 mM glutamine, 436.9 U/mL penicillin, 0.4 mg/mL streptomycin, 2.2 µg/mL amphotericin B, and 8.7% fetal bovine serum) using fire-polished Pasteur pipettes. The mesencephalic cells were plated on poly-D-lysine-coated 48-well plates (Greiner, Bio-One, Kremsmünster, Austria) (density 255,000 cells in 340 µL per well) and were grown in an incubator at 37 °C, 5% CO_2_, and 100% humidity. The culture medium was exchanged on the 1st day in vitro (DIV) and the 3rd DIV. On the 5th DIV, half of the medium was replaced by a serum-free B27-supplemented medium (DMEM without pH indicator supplemented with 9.4 mM HEPES, 10.4 mM glucose, 1.9 mM glutamine, 472 U/mL penicillin, 0.5 mg/mL streptomycin, 1.4% B27 supplement (Gibco, Carlsbad, CA, USA)). From the 6th DIV onwards, a serum-free B27-supplemented medium was used and changed every 2nd day.

### 2.2. Treatment of Dissociated Mesencephalic Cell Cultures

TQ and rotenone were dissolved in dimethyl sulfoxide (DMSO). MPP^+^ was directly dissolved in a serum-free B27-supplemented medium and the final concentrations were prepared. The DMSO concentration in the culture medium did not exceed 0.01%. In order to analyze the possible effects of TQ, cell cultures were treated with TQ in different concentrations (0.01, 0.1, 1, 10 µM) on the 10th DIV for 48 h. To investigate the potential neuroprotective effects of TQ on the dopaminergic neurons, cell cultures were concomitantly treated with rotenone (20 nM) or MPP^+^ (10 µM) and TQ (0.01, 0.1, 1, 10 µM) on the 10th DIV for 48 h.

### 2.3. Cultivation of Cell Line N18TG2

N18TG2 neuroblastoma cells (DSMZ, Braunschweig, Germany) were kept in 25 to 75 cm^2^ cell culture flasks (Greiner, Bio-one, Kremsmünster, Austria) in an incubator at 37 °C, 5% CO_2_, and 100% humidity and were split (1:10) when they reached approximately 80% confluence (2–3 times per week). The neuroblastoma medium contained DMEM with 9.7% fetal bovine serum, 1.94 mM glutamine, and 1.94 mM sodium pyruvate.

### 2.4. Treatment of Neuroblastoma Cells

The treatment of the neuroblastoma cells was performed in 48-well or 96-well plates (Greiner, Bio-One, Kremsmünster, Austria) (clear, flat bottomed, coated with poly-D-lysine). The cells were collected by centrifugation (10 min at 178 g) and resuspended in DMEM without a pH indicator (supplemented with 1.94 mM glutamine, 1.94 mM sodium pyruvate, and 2% of B27 supplement) in a final concentration of 3 × 10^5^ cells/mL. The cell cultures were treated with TQ in different concentrations (0.01, 0.1, 1, 10 µM) for 48 h. To investigate the potential effects of TQ, the cell cultures were concomitantly treated with rotenone (160 nM).

### 2.5. Anti-Tyrosine Hydroxylase Immunocytochemistry

On the 12th DIV, cultures were fixed with 4% paraformaldehyde (20 min at 4 °C) and permeabilized with 0.4% Triton-X solution (30 min at room temperature). Between the following steps, the cultures were washed twice with PBS. To block the nonspecific binding sites, the cells were incubated in 5% horse serum. Thereafter, the cells were incubated with the anti-tyrosine hydroxylase antibody (1:1000 in DPBS (Millipore, Darmstadt, Germany)) at 4 °C overnight. Then, the cells were incubated with the biotinylated secondary antibody for 90 min at room temperature, followed by incubation with the avidin biotin horseradish peroxidase complex, according to manufacturer’s protocol (ABC Kit Vectastain, Burlingame, CA, USA). The immunoreaction was visualized using diaminobenzidine and H_2_O_2_. Finally, the cells were covered with Kaiser’s glycerol gelatine. The total number of tyrosine-hydroxylase immunoreactive cells was evaluated in 18–22 fields/well, using a Nikon inverted microscope at 100× magnification. For the measurement of the neurite lengths of the dopaminergic cells, photographs of six representative cells per condition for each experiment were taken. The measurement of the neurite lengths was performed using Adobe Photoshop^®^ CS3 and a standard scale was used as reference. For this purpose, the quantity of pixels used for manually tracing the neurite lengths was compared to the pixels used for 100 µm of the reference scale. The calculations were performed using Microsoft Excel software (version 365).

### 2.6. Resazurin Reduction Test

After incubation of the primary mesencephalic and N18TG2 neuroblastoma cells with TQ ± rotenone, resazurin (45 µM) was added to the culture medium. The time-dependent reduction was assessed after 0 and 3 h by measuring the absorbances at 570 and 600 nm (Tecan Sunrise, Tecan, Männedorf, Switzerland). The cell cultures were kept at 37 °C and 5% CO_2_ between measurements. The reduction of resazurin to resorufin was calculated as follows:

$$\left[\frac{\left(O_{2}\times A_{1}-O_{1}\times A_{2}\right)}{\left(R_{1}\times N_{2}-R_{2}\times N_{1}\right)}\right]\times 100$$

*O*_1_ is the molar extinction coefficient (ε) of resazurin at 570 nm (80.586); *O*_2_, ε of resazurin at 600 nm (117.216); *R*_1_, ε of resorufin at 570 nm (155.677); *R*_2_, ε of resorufin at 600 nm (14.652); *A*_1_ is the absorbance of the test probes at 570 nm; *A*_2_ is the absorbance of the test probes at 600 nm; *N*_1_ is the absorbance of the negative control (no cells) at 570 nm; *N*_2_ is the absorbance of the negative control (no cells) at 600 nm. The reduction of resazurin to resorufin in the vehicle control was set to 100%.

### 2.7. Determination of NO Production

NO radical detection was performed using 4-amino-5-methylamino-2′,7′-difluorofluorescein diacetate (DAF-FM diacetate, Invitrogen, Carlsbad, CA, USA). The cultured mesencephalic cells were incubated with DAF-FM diacetate (10 µM in DMEM without pH indicator, 1 h at 37 °C). In cells, DAF-FM diacetate is deacetylated by esterases to the nonfluorescent DAF-FM, which then reacts with NO to form fluorescent benzotriazole and provide diffuse staining. After incubation with fluorescence dye, the photographs were taken by a camera (Nikon, Japan) attached to an inverted microscope with epifluorescence equipment. The colour intensities for each photo were analysed by Adobe Photoshop^®^ CS3 software. The averaged colour intensity was measured with the histogram modus in the regions of interest (1.68 mm^2^/field).

### 2.8. Superoxide Measurement Using Dihydroethidium and Live-Cell Microscopy

In the presence of superoxide radicals, dihydroethidium (DHE, Invitrogen, Carlsbad, CA, USA) is intracellularly oxidised to ethidium, which fluorescens when intercalated into the DNA. The DHE staining was performed by adding DHE solution (10 µM in DMEM without a pH indicator) to unfixed cells and incubation for 30 min at 37 °C. After incubation with fluorescence dye, the photographs were taken by a camera (Nikon, Tokio, Japan) attached to an inverted microscope with epifluorescence equipment and analysed by Adobe Photoshop^®^ CS3 software. The averaged colour intensity was measured with the histogram modus in the regions of interest (1.68 mm^2^/field).

### 2.9. Superoxide Measurement Using Dihydroethidium and Flow Cytometry

N18TG2 neuroblastoma cells were seeded in 24-well plates (clear, flat bottomed) at a concentration of 10^5^ cells/well, and 750 µL cell suspension/well. At the end of the treatment incubation period, a final concentration of 10 µM of DHE in DMEM without a pH indicator was incubated for 30 min before the cells were dissociated using a cell strainer and, finally, collected in FACS tubes. The fluorescence measurement data were acquired on a FACScan (BD Biosciences, San Jose, CA, USA) flow cytometer equipped with a 488 nm argon laser. For analysis, the BD CellQuest Suite Version 3.1f was used. The minimum event count was set to 10^4^ events, measured at a high flow rate (60 µL/min). Differentiation on forward- and sideward-scatter of events was performed by manual gating. The fluorescence of the DHE was evaluated at 530 nm using the FL-2 channel in histogram mode. The median values were calculated using the built-in software functions.

### 2.10. Quantification of Superoxide Radicals by Electron Paramagnetic Resonance (EPR)

EPR spectroscopy is based on the absorption of microwave radiation stimulated by an electromagnetic field by free radicals and unpaired electrons. O_2_^●−^ radicals are instable but form stable nitroxyl radicals with 1-hydroxy-3-methoxycarbonyl-2,2,5,5-tetramethylpyrrolidine (CMH), which can be detected by EPR [21]. N18TG2 neuroblastoma cells were collected by centrifugation (10 min at 178 g) and resuspended in a Ringer solution with a concentration of 2 × 10^6^ cells/mL. Each condition (TQ (10 µM) ± rotenone (500 nM)) was measured every minute for 10 min. For every sample, three scans were accumulated. The instrument settings were: 9.684 GHz microwave frequency, 3449 G (Gauss magnetic field strength) centre field, 80 G sweep, 1 G modulation amplitude, 2 × 10^4^ receiver gain, and 0.082 s time constant. The formation of oxidised CMH was quantified in pmol/min and the previously measured controls were set to 100%.

### 2.11. Monitoring of the Mitochondrial Membrane Potential (ΔΨm) via JC-1

The ΔΨm of the intact cells was monitored by an inverted fluorescence microscope with the lipophilic probe JC-1. At the end of the TQ/rotenone incubation, the culture medium was removed and, then, the cells were loaded for 0.5 h at 37 °C with JC-1 (0.1 µg/mL (molecular probes, Eugene, OR, USA)), which accumulates as J aggregates in mitochondria by an electrochemical gradient, indicated by a fluorescence emission shift from green (~529 nm) to red (~590 nm). Mitochondrial depolarization is allocated by a decrease in the red/green fluorescence intensity ratio. The JC-1 monomers detection was performed at 485 nm and 535 nm as excitation and emission wavelength, respectively. The J aggregates were measured using 485 nm excitation and 590 nm emission, respectively. After incubation with fluorescence dye, the photographs were taken by a camera (Nikon, Tokio, Japan) attached to an inverted microscope with epifluorescence equipment. The colour intensities for each photo were analysed by Adobe Photoshop^®^ software. The averaged colour intensity was measured with the histogram modus in the regions of interest (1.68 mm^2^/field).

### 2.12. ATP Measurements

The ATP levels were determined using a conventional luciferase ATP kit (Promega, Medison, WI, USA). The N18TG2 neuroblastoma cells were seeded at a final density of 4 × 10^3^ cells/well on a 96-well plate. The primary cells were plated at a density of 25.5 × 10^4^ cells/well on a 48-well plate. A CellTiter Glo^®^ reaction solution was added after medium removal, incubated on an orbital shaker for 30 min at room temperature, and measured by an EnSpire^®^ (PerkinElmer, Waltham, MA, USA) luminescence reader.

### 2.13. Determination of Glutathione Level

The total intracellular reduced glutathione (GSH) and total glutathione (glutathione disulfide (GSSG + GSH) were measured using a glutathione detection kit (Cayman Chemical, Ann Arbor, MI, USA), according to the manufacturer’s instructions. Briefly, the primary mesencephalic and N18TG2 cells were rinsed twice with PBS and, then, sonicated in 400 µL MES buffer. After centrifugation (12,100× *g* for 15 min), the cell lysates were mixed with a freshly prepared assay cocktail. This cocktail contained glutathione reductase with or without 2-vinylpyridine to discriminate between total glutathione and GSH. Absorbance was measured at 405 nm using a microtiter plate reader (PerkinElmer, Waltham, MA, USA).

### 2.14. Determination of Caspase-3 Activity

Caspase-3 activity was measured using a caspase-3 colorimetric assay kit (Sigma Aldrich, Munich, Germany). After treatment, the primary mesencephalic and N18TG2 cells were lysed by adding 100 µL lysis buffer (30 min, 4 °C). Subsequently, to separate the cell fragments, the cell lysate was centrifuged at 12,100× *g* for 15 min at room temperature. The supernatants were incubated with assay buffer and substrate, according to the manufacturer’s instructions. The generation of p-nitroaniline was measured at 405 nm, using a microtiter plate reader (PerkinElmer, Waltham, MA, USA). The caspase-3 activity was calculated from the absorbance value using a calibration curve, and an increase in caspase-3 activity was expressed as a percentage of the control values.

### 2.15. Statistics

The calculations for the statistical differences were performed using the nonparametric Kruskal–Wallis test followed by a Chi square (χ^2^) test. For the evaluation of the rotenone/MPP^+^ effects vs. the control, the nonparametric Mann–Whitney U test was used. The *p* < 0.05 was considered as statistically significant.

## 3. Results

### 3.1. Effects of TQ on the Number of THir Cells under Oxidative Stress Conditions

**Figure 1:**
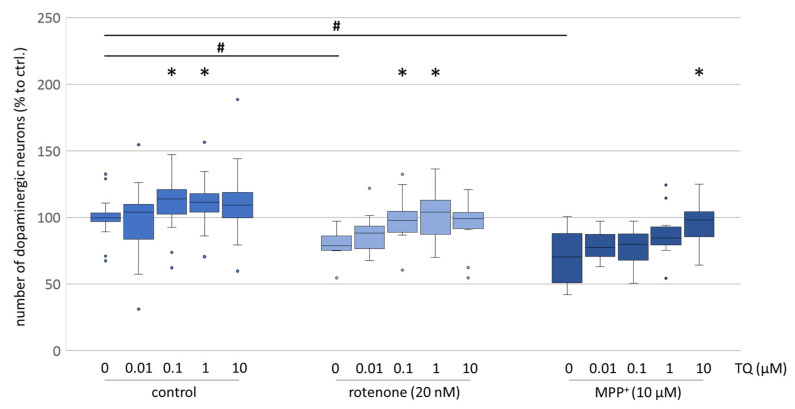
Effects of TQ on the number of THir (dopaminergic) neurons in rotenone- or MPP^+^-affected primary mesencephalic cultures after 48 h treatment. Specifically, 100% corresponds to the number of dopaminergic neurons in the untreated controls (0 µM TQ, 0 nM rotenone, 0 µM MPP^+^). The data represent at least five independent experiments. Statistically significant differences between the TQ groups compared to the respective controls were determined using the Kruskal–Wallis (H) test followed by the χ^2^ test (* *p* < 0.05). The Mann–Whitney (U) test was used to evaluate the effects of rotenone/MPP^+^ vs. the control (# *p* < 0.05).

Dopaminergic neurons contribute approximately 0.1% of the total cell population in primary mesencephalic cultures. In control cultures, the absolute number of dopaminergic neurons was 635 ± 85 cells per well. TQ treatment significantly enhanced the survival of dopaminergic neurons at 0.1 and 1 µM by 14.2 and 11.7%, respectively (Figure 1). While rotenone exposure decreased the number of dopaminergic neurons to 78.6%, co-treatment with TQ protected the dopaminergic neurons significantly, reaching the control values at 0.1 and 1 µM of TQ (Figure 1). The exposure of mesencephalic cell cultures to MPP^+^ lowered specifically and significantly the number of dopaminergic neurons to 70.3%. A recovery in the survival of MPP^+^-treated dopaminergic neurons to the number of untreated control cells was observed in the presence of the highest tested TQ concentration (Figure 1).

### 3.2. Effects of TQ on the Length of Primary Neurites under Oxidative Stress Conditions

**Figure 2:**
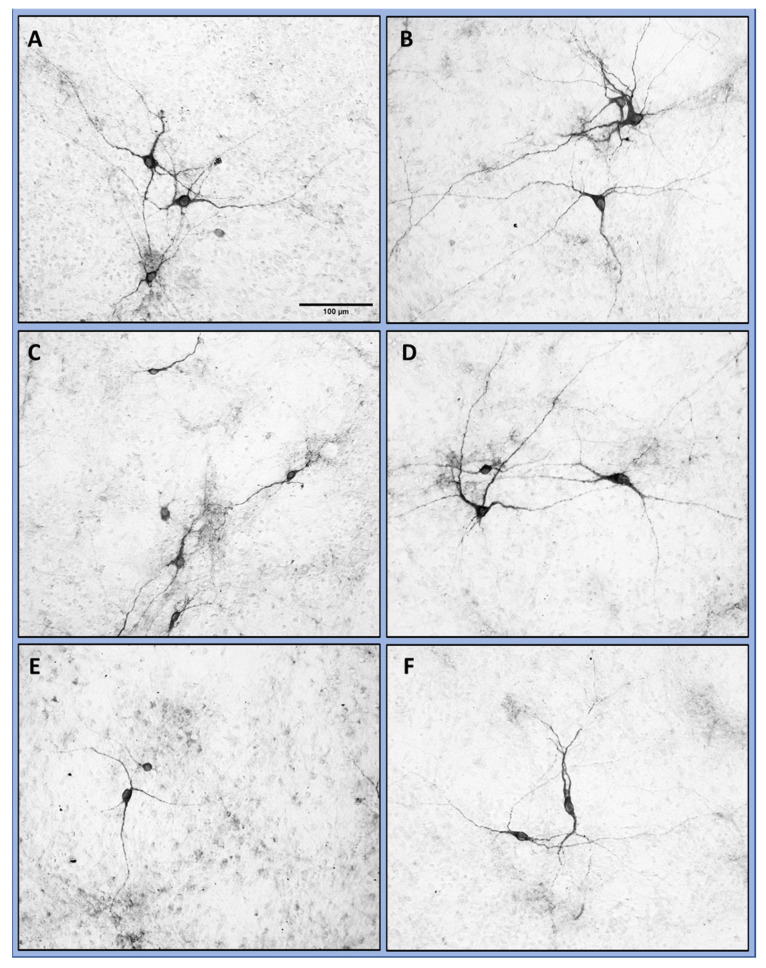
Representative photographs of the THir neurons under different conditions. (**A**) Neurons in the untreated controls (0 µM TQ, 0 nM rotenone, 0 µM MPP^+^). (**B**) Neurons treated with 10 µM TQ. (**C**) Tyrosine hydroxylase positive neurons treated with 10 µM MPP+. (**D**) Cells concomitantly treated with 10 µM TQ and 10 µM MPP^+^. (**E**) Dopaminergic neurons treated with 20 nM rotenone. (**F**) THir neurons concomitantly treated with 10 µM TQ and 20 nM rotenone.

Not only was the number of THir (dopaminergic) neurons reduced by MPP^+^ and rotenone, but the morphology also changed (Figure 2). The cell bodies were shrunk, and the neurite number was reduced. These changes could be partially counteracted by TQ (Figure 2). To evaluate these changes, the neurite lengths were measured.

**Figure 3:**
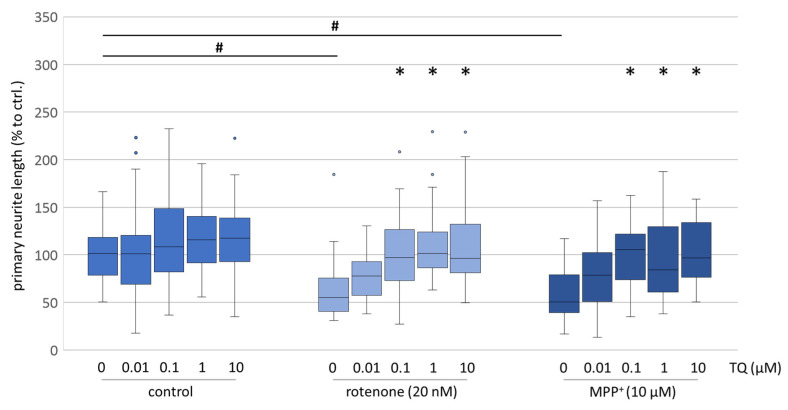
Effects of TQ on the length of the primary neurites of THir (dopaminergic) neurons in rotenone- or MPP^+^-affected primary mesencephalic cultures after 48 h treatment. Specifically, 100% corresponds to the number of dopaminergic neurons in the untreated controls (0 µM TQ, 0 nM rotenone, 0 µM MPP^+^). The data represent at least three independent experiments. Statistically significant differences between the TQ groups and the respective controls were determined using the Kruskal–Wallis (H) test followed by the χ^2^ test (* *p* < 0.05). The Mann–Whitney (U) test was used to evaluate the effects of the rotenone/MPP^+^ vs. the control (# *p* < 0.05).

Neurite outgrowth is known as an energy-consuming process, thus, the measurement of cell morphology is a useful tool to evaluate the health status of dopaminergic neurons. After exposure to rotenone, the neurite lengths of dopaminergic neurons were significantly shortened to 55.1%, when compared to the control (Figure 3). The concomitant administration of TQ was able to preserve the morphology of the cells and mitigate the neurotoxic effects of rotenone significantly at the TQ concentrations of 0.1, 1, and 10 µM, when compared to the untreated control (Figure 3). The administration of MPP^+^ significantly decreased the neurite length to 50.5%, while the co-treatment with TQ led to a concentration-dependent increase in the neurite length nearly to the control values, with significance at 0.1, 1, and 10 µM TQ (Figure 3).

### 3.3. Effects of TQ on the Formation of Resorufin ± Rotenone

**Figure 4:**
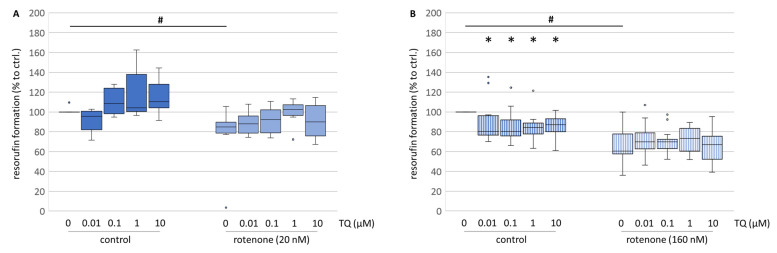
Effects of TQ on the formation of resorufin in primary mesencephalic cultures (**A**) and the neuroblastoma cell line (**B**) in the absence and presence of rotenone after 48 h treatment. The untreated control groups (0 µM TQ, 0 nM rotenone) were set to 100%. The data represent six (**A**) or ten (**B**) independent experiments. Statistically significant differences between TQ groups compared to the respective controls were determined using the Kruskal–Wallis (H) test followed by the χ^2^ test (* *p* < 0.05). The Mann–Whitney (U) test was used to evaluate the effects of rotenone vs. the control (# *p* < 0.05).

The formation of resorufin was perceptibly but not significantly higher in the TQ-treated mesencephalic cell culture (Figure 4A). Treatment with rotenone lowered the reduction of resazurin to resorufin, significantly, to 85.0% compared to the untreated control (Figure 4A). The concomitant administration of rotenone and TQ in the primary mesencephalic cell culture resulted in no significant enhancement of the resorufin formation (Figure 4A). Nonetheless, treatment with TQ at a concentration of 1 µM mitigated the effect of rotenone and increased the resorufin formation nearly to the control level.

Unlike in mesencephalic cell cultures, treatment with TQ significantly reduced the formation of resorufin in the neuroblastoma cell line in all tested concentrations (0.01, 0.1, 1, and 10 µM) to 80.3, 80.2, 84.3, and 87.1%, respectively, compared to the untreated control (Figure 4B). While rotenone treatment significantly decreased the formation of resorufin to 60.5%, co-treatment with TQ did not re-establish the reduction of resazurin in the neuroblastoma cell line (Figure 4B).

### 3.4. Effects of TQ on the NO or ROS Content

**Figure 5:**
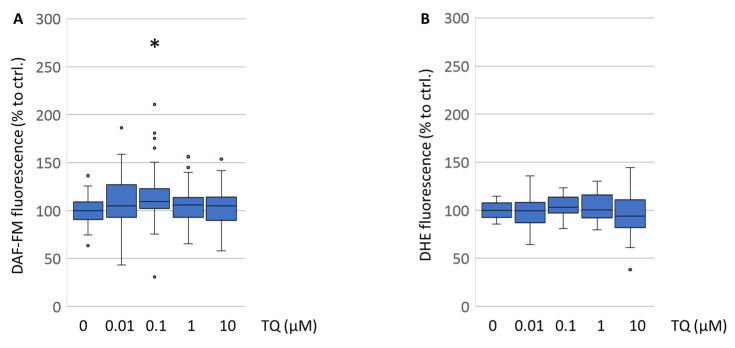
NO concentrations (**A**) and ROS formation (**B**) of 48 h TQ-treated primary mesencephalic cultures. Untreated control groups were set to 100%. The data represent seven independent experiments. Statistically significant differences between the TQ groups comoared to the respective controls were determined using the Kruskal–Wallis (H) test followed by the χ^2^ test (* *p* < 0.05).

Evaluation of the NO content in the primary mesencephalic cell culture using the DAF-FM staining showed a moderately, although significantly, increased NO level at 0.1 µM TQ (Figure 5A). The measurement of the ROS concentrations in the primary mesencephalic cell culture revealed no significant changes for all the tested concentrations of TQ (Figure 5B).

### 3.5. Influence of TQ on ROS Formation under Oxidative Stress Conditions

**Figure 6:**
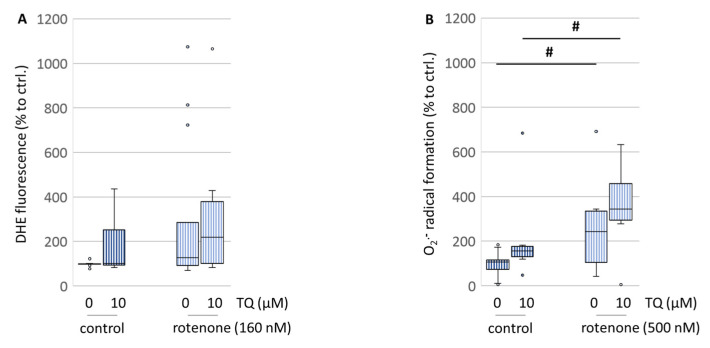
Effects of TQ on ROS formation detected by DHE fluorescence (48 h treatment) (**A**) and EPR spectroscopy (immediately after exposer) (**B**) in neuroblastoma cell line in the absence and presence of rotenone. The untreated control groups (0 µM TQ, 0 nM rotenone) were set to 100%. The data represent four (**A**) or eight (**B**) independent experiments. Statistical significance was determined using the Mann–Whitney (U) test (# *p* < 0.05).

EPR spectroscopy data and flow cytometry data of the DHE-stained cells revealed that rotenone increased superoxide radical formation, but significant changes were observed only in the EPR data, even reaching an increase to 243.5%, when compared to the control (Figure 6B). TQ did not significantly reduce the formation of superoxide radicals, when measured with both methods (Figure 6A,B). On the contrary, a tendency toward a further increase in superoxide radical formation after TQ administration was observed. The EPR data revealed a significantly higher superoxide radical formation, even to 343.7%, when compared to the control, in cultures concomitantly treated with rotenone and TQ (Figure 6B).

### 3.6. Effects of TQ on ΔΨm in Rotenone Affected Cells

**Figure 7:**
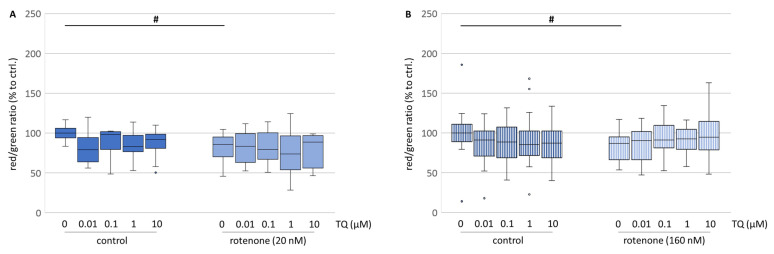
Effects of TQ on the mitochondrial membrane potential of the primary mesencephalic cultures (**A**) and the neuroblastoma cell line (**B**) in the absence and presence of rotenone after 48 h treatment. The untreated control groups (0 µM TQ, 0 nM rotenone) were set to 100%. The data represent five (**A**) or eight (**B**) independent experiments. Statistical significance was determined using the Mann–Whitney (U) test (# *p* < 0.05).

After exposure of the primary mesencephalic cell culture to rotenone, the mitochondrial membrane potential was significantly lowered by 14.1%, compared to the control cultures (Figure 7A). TQ in all the tested concentrations led to a similar, non-significant tendency to decrease the mitochondrial membrane potential (Figure 7A). The cultures treated concomitantly with rotenone, causing impaired mitochondrial membrane potential, did not show any beneficial effect of TQ (Figure 7A).

Treatment of the neuroblastoma cell line with TQ alone, also resulted in a tendency toward lowered mitochondrial membrane potential (Figure 7B). Rotenone alone reduced the mitochondrial membrane potential significantly by 13.0% (Figure 7B). The co-treatment of the neuroblastoma cell line with TQ and rotenone, led to a non-significant, concentration-dependent tendency of TQ to restore the mitochondrial membrane potential (Figure 7B).

### 3.7. Effects on the ATP Level after Treatment with TQ ± Rotenone

**Figure 8:**
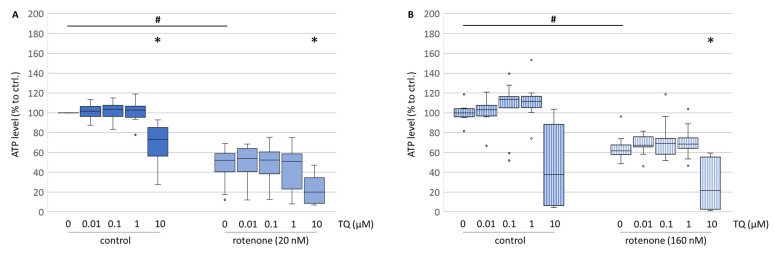
Effects of TQ on the total ATP level in primary mesencephalic cultures (**A**) and the neuroblastoma cell line (**B**) after 48 h treatment. The untreated control groups (0 µM TQ, 0 nM rotenone) were set to 100%. The data represent six (**A**) or ten (**B**) independent experiments. Statistically significant differences between the TQ groups compared to the respective controls were determined using the Kruskal–Wallis (H) test followed by the χ^2^ test (* *p* < 0.05). The Mann–Whitney (U) test was used to evaluate the effects of rotenone vs. the control (# *p* < 0.05).

The administration of TQ did not exhibit any effect on the ATP level in the primary cell culture except at the highest concentration of 10 µM TQ, which reduced the ATP level significantly to 73.1% (Figure 8A). The exposure of the mesencephalic cell culture to rotenone resulted in a significantly lowered ATP level (52.0%) (Figure 8A). Co-treatment of the primary cell culture with rotenone and TQ did not alter the ATP level, when compared to the exposure to rotenone alone, with the exception of 10 µM of TQ, when a significantly lowered ATP level to 20.0% was observed (Figure 8A).

Treatment of the neuroblastoma cell line with TQ led to a similar result. Treatment with TQ alone had no significant effect on the ATP level (Figure 8B). However, TQ at a concentration of 10 µM showed a non-significantly lowered ATP level to 37.9%, in comparison to the control (Figure 8B). The administration of rotenone reduced the ATP level significantly to 61.7%, compared to the untreated control (Figure 8B). The co-treatment with rotenone and TQ showed, exclusively at 10 µM of TQ, a significantly lowered ATP level of 21.6% (Figure 8B).

### 3.8. Glutathione Levels of TQ and Rotenone Affected Cells

**Figure 9:**
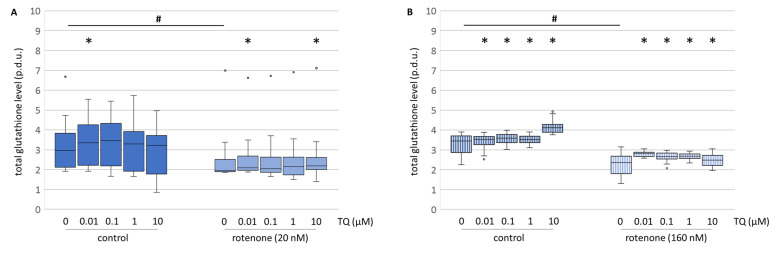

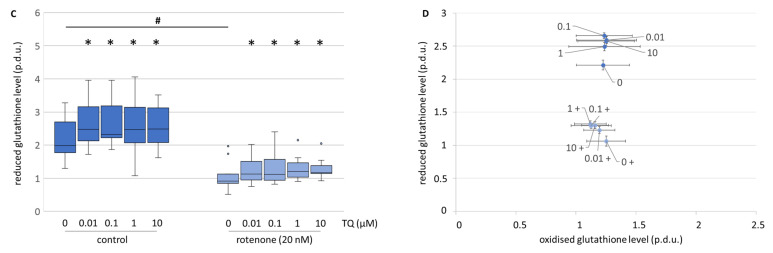
Effects of TQ on the total glutathione (GSSG + GSH) and reduced (GSH) levels in primary mesencephalic cultures (**A**,**C**) and total glutathione (GSSG + GSH) levels in the neuroblastoma cell line (**B**) and a comparison of reduced/oxidised glutathione (**D**) in the absence and presence of rotenone after 48 h treatment. The Data represent 14 (**A**), 4 (**B**), or 9 (**C**) independent experiments. Statistically significant differences between the TQ groups compared to the respective controls were determined using the Kruskal–Wallis (H) test followed by the χ^2^ test (* *p* < 0.05). The Mann–Whitney (U) test was used to evaluate the effects of rotenone vs. the control (# *p* < 0.05).

In mesencephalic cell cultures, the levels of total glutathione (GSSG + GSH) and reduced glutathione (GSH) was found to be above the control level in all tested TQ concentrations, with a statistically significant increase in the total glutathione level for 0.01 µM TQ to 3.4 p.d.u. (Figure 9A) and an increased level of reduced glutathione to 2.5, 2.3, 2.5 and 2.5 p.d.u. for 0.01, 0.1, 1, and 10 µM TQ, respectively (Figure 9C,D).

Exposure of the mesencephalic cell culture to rotenone decreased the total and the reduced glutathione levels significantly to 1.9 p.d.u. (Figure 9A) and 0.9 p.d.u. (Figure 9C), when compared to the control. The data on the concomitant treatment of the primary cell culture with TQ and rotenone, displayed a tendency to re-establish both total and reduced glutathione levels in all tested concentrations (Figure 9A,C,D), with a significant increase in the total glutathione level to 2.1 and 2.2 p.d.u. at 0.01 and 10 µM TQ, respectively.

In the neuroblastoma cell line treated with TQ, the total glutathione level was shown to increase in a dose-dependent manner, with statistical significance at all tested concentrations of TQ to 3.5, 3.6, 3.5, and 4.1 p.d.u., when compared to the untreated culture (Figure 9B).

Exposure of the neuroblastoma cell line to rotenone significantly decreased the total glutathione level to 2.3 p.d.u., when compared to the control (Figure 9B). After co-treatment with TQ, a statistically significant restoring effect on the total glutathione level was observed for TQ at all the tested concentrations (Figure 9B), causing an increase to 2.8, 2.7, 2.7, and 2.5 p.d.u., respectively, when compared to the control.

### 3.9. Caspase-3 Activity Influenced by TQ ± Rotenone

**Figure 10:**
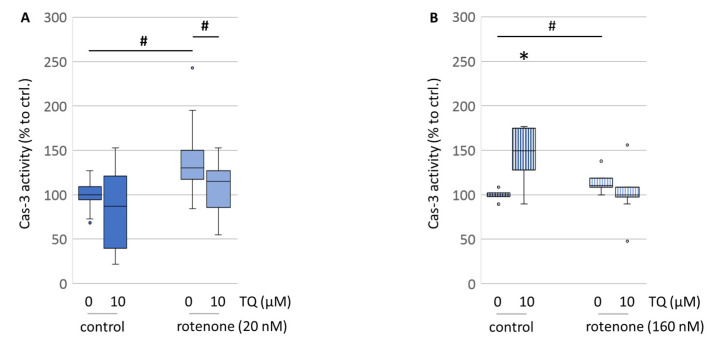
Effects of TQ on caspase-3 activity in primary mesencephalic cell culture (**A**) and the neuroblastoma cell line (**B**) in the absence and presence of rotenone after 48 h treatment. The untreated control groups (0 µM TQ, 0 nM rotenone) were set to 100%. The Data represent nine (**A**) or four (**B**) independent experiments. Statistically significant differences between the TQ group compared to the respective controls were determined using the Kruskal–Wallis (H) test followed by the χ^2^ test (* *p* < 0.05). Statistical significance was determined using the Mann–Whitney (U) test (# *p* < 0.05).

In the primary mesencephalic cell culture, TQ was shown to have a mild lowering effect on caspase-3 activity, and significantly reduced its activity in rotenone co-treated cultures, while after exposure to rotenone alone, an increase in caspase-3 activity by 30.2% was observed (Figure 10A).

A massive pro-apoptotic effect of TQ in the neuroblastoma cell line could be observed with a significantly increased caspase-3 activity by 49.7%, when compared to the control (Figure 10B). Treatment with rotenone tended to result in a moderate, but significantly increased, caspase-3 activity, which was unaffected by a co-treatment with TQ (Figure 10B).

## 4. Discussion

This study was designed to answer the question on whether TQ is likely to rescue neurons in an oxidative stress model through antioxidative, or even radical scavenging, mechanisms. Thymoquinone, a promising active compound from *Nigella sativa* seeds, has been postulated to have many pharmacological properties related to cell protection under oxidative stress conditions [22,23,24]. According to the findings of Radad et al., treatment with TQ led to a significant increase in the survival rate of dopaminergic neurons [9]. Our findings confirmed the neuroprotective effect of TQ using tyrosine hydroxylase immunocytochemistry (Figure 1), showing an increased survival rate of dopaminergic neurons exposed to oxidative stress (MMP^+^/rotenone). The evaluation of neurite outgrowth, which is a high energy-consuming process and a reliable indicator of the energetic condition of cells, likewise showed that TQ is able to mitigate the neurotoxic effect of rotenone (Figure 2 and Figure 3). Additionally, the neurotoxic effect of the complex I inhibitor, MPP^+^, which acts specifically on dopaminergic neurons, was decreased when TQ was co-administrated (Figure 1). Moreover, in our cellular PD model, the morphology of the cells was preserved (Figure 2) and the neurite outgrowth was less diminished (Figure 2 and Figure 3). These findings are consistent with the neuroprotective effects of TQ, previously postulated by other groups investigating several models, like cisplatin-induced neurotoxicity, the oxidation of amyloid beta (Aß) or the effects of rotenone on the dopaminergic neurons in the substantia nigra [15,16,17]. To elucidate the mode of action of TQ further, we determined the metabolic activity in our cell culture models.

Primary culture treated with TQ confirms the promising data obtained from immunocytochemistry, especially the neurite outgrowth. Administration of TQ alone increased the metabolic activity (Figure 4A), while when co-administrated with rotenone, TQ was able to improve cell metabolism nearly up to the control levels (Figure 4A). Although this increase was not significant, a stabilising effect of TQ on the cell metabolism might be involved. On the contrary, in neuroblastoma cells, TQ reduced the formation of resorufin in all used concentrations (Figure 4B). Since neuroblastoma cells are still dividing cells in culture, measurement of the formation of resorufin in this model reflects more the cell number than the metabolic activity. Hence, our data are in accordance with the findings of Mostofa and his colleagues, who postulated TQ as a substance with broad anti-cancer properties [6].

The antioxidative or superoxide radical scavenging hypothesis proposed by Badary et al. is a widespread explanation for the general beneficial effects of TQ [10]. The hypothesis that TQ is a potent superoxide radical scavenger has been supported by several authors [10,18]. TQ has been also suggested to act as a superoxide scavenger, with an effectiveness comparable to superoxide dismutase [10]. Moreover, Nagi and Mansour reported that TQ has protective effects against doxorubicin-induced cardiotoxicity in rats, which is thought to be induced by superoxide radicals [25]. Consequently, we reassessed the influence of TQ on nitric oxide status in the cells on DAF-FM and ROS formation detected by DHE fluorescence measurements. Interestingly, TQ had no effect on the levels of reactive oxygen species in the primary mesencephalic cell culture (Figure 5B). Nitric oxide levels were even significantly augmented by TQ (Figure 5A). Since our data obtained from the primary cells are quite contradictory to those findings, we investigated the superoxide radical scavenging capacities of TQ using more sensitive parameters like flow cytometry and EPR spectroscopy, which is to date the most selective and quantitative method for observations on radical formation. For both methods, the N18TG2 neuroblastoma cells were used because of the inaccessibility of the primary cell culture for those methods. The EPR spectroscopy data showed that TQ itself increased the superoxide radical formation immediately after treatment (Figure 6B). The flow cytometry data from the DHE-stained cells showed that TQ did no reduce the overall formation of ROS after treatment. On the contrary, a further increase was observed (Figure 6A). However, TQ was not able to counteract this action resulting even in higher ROS formation (Figure 6B). Thus, our findings suggest that TQ is not operating via its superoxide radical scavenging properties. In contrast, it appears to enhance the formation of superoxide radicals. This is in accordance with the findings of El-Najjar and colleagues, who reported that TQ exhibits an anti-proliferative effect on colon cancer cells via the generation of ROS and, consequently, on the activation of the JNK/ERK pathway [26].

Superoxide radical formation in the cell might be accompanied by modulation of the mitochondria activity and modification of the integrity of the mitochondrial membranes [27]. Interestingly, evaluation of the membrane potential of mitochondria (ΔΨ_m_) after treatment with TQ revealed a mild depolarisation in both cell culture systems (Figure 7A,B). This might be a result of the high lipid solubility of TQ. Roepke and his colleagues stated that the incorporation of TQ into the inner mitochondrial membrane led to electron leakage [28]. In contrast, Vyssokikh and his colleagues reported that a decrease in ΔΨ_m_ was not necessarily connected with an impairment of the mitochondria [29]. This is partially consistent with our findings, where treatment with TQ caused a mild depolarisation of the ΔΨ_m_ (Figure 7A,B), but immunocytochemistry showed an increased survival of the dopaminergic neurons. In contrast, in the neuroblastoma cell line the depolarization might be associated with the lowered cell number revealed by resorufin formation. Korshunov and his colleagues stated that the lowered ΔΨ_m_ may result in a complete inhibition of the formation of mitochondrially derived reactive oxygen species, while the ATP formation remains unaffected [30]. Therefore, we evaluated the ATP level in both cell culture systems.

Except at the highest concentration of TQ, in mesencephalic cells the ATP levels were mostly unaffected by TQ alone (Figure 8A), while in the neuroblastoma cell culture TQ had a tendentially increasing effect on the ATP level (Figure 8B). However, concomitant treatment with TQ and rotenone had no restoring effect on the ATP levels (Figure 8A,B). Thus, TQ-mediated neuroprotection is not mediated via restoration of the ATP supply and the mild depolarisation of mitochondria does not affect the ATP formation. Thereby, the lowered resorufin formation, representing neuroblastoma cell numbers, in all concentrations might not be a result of lowered ATP production. Only at a TQ concentration of 10 µM were ATP levels lowered, which might point to another energy-consuming process induced by TQ, such as the induction of autophagy and/or apoptosis [31].

While these data do not explain TQ’s neuroprotective characteristics, TQ might indirectly increase the antioxidative capacity, through mechanisms that involve the transcriptional and post-transcriptional regulation of antioxidants, like the modification of glutathione via the Nrf2-Keap1 pathway [32].

TQ, in all the tested concentrations, moderately elevated the level of total glutathione in the mesencephalic cell culture (Figure 9A). The differentiation between the reduced and the oxidised form of glutathione showed that the elevated level of total glutathione was due to a higher level of the reduced form (Figure 9C,D). Rotenone decreased the total glutathione level and the reduced form to the same extent (Figure 9A,C). Co-treatment with TQ and rotenone caused a dose-dependent and comparable increase in the total, as well as the reduced form (Figure 9A,C). Similar effects on the total glutathione level were observed in the neuroblastoma cell line (Figure 9B). This is in accordance with Khalife and Lupidi, who stated that TQ may contribute to a more effective endogenous anti-oxidant defence system [33]. This is proposed to happen through an enzymatic and non-enzymatic reaction of TQ to thymohydroquinone and glutathionyl-dihydro thymoquinone. Nonetheless, it has to be taken into consideration, that the reduction of TQ into its metabolites is a process that leads to the formation of cytosolic ROS [34]. This might be an explanation as to why no decrease in ROS levels were found after TQ administration. Additionally, it was shown that tert-butyl hydroxyquinone directly interacts with the Nrf2-Keap1 pathway. This leads to the induction of genes involved in the coordinated response to oxidative stress and, consecutively, to restored cellular redox homeostasis and diminished oxidative damage [32]. The interaction by TQ with glutathione via the Nrf2-Keap1 pathway might be a mode of action even though we did not test it. It remains for a further study to clarify this aspect. Nonetheless, the heightened level of reduced glutathione might result in a higher survival rate of dopaminergic neurons, but does not explain the lowered resorufin formation in neuroblastoma cells.

Berker et al. stated that TQ decreased the expression levels of caspase-3 in healthy hippocampal tissue and significantly increased the number of hippocampal neurons [35]. In our study, caspase-3 activity was significantly lowered by TQ in the rotenone-treated primary mesencephalic cell culture, while TQ alone was shown to have a mild lowering effect on its activity (Figure 10A), which is consistent with their findings. Together with a larger level of reduced glutathione, this might be an explanation for the higher cell survival of the dopaminergic neurons under oxidative stress conditions. In contrast, in the neuroblastoma cell line, TQ alone had an impact on caspase-3, causing a 1.5-fold increase in its activity, when compared to the untreated control (Figure 10B). This extensive shift towards a pro-apoptotic condition might be an explanation for the lowered resorufin formation and the reduced ATP levels. This might be indicative of the hypothesized mechanism of the anti-cancer properties of TQ [36].

## 5. Summary

Considering the high number of publications about thymoquinone, the impression that it could be a “panacea” with a high antioxidative potential is given. Nonetheless, the chemical synthesis of TQ in the plant via oxidations makes it inapplicable for scavenging radicals. The reassessment of the superoxide radical scavenging properties of TQ revealed that other modes of action must be involved. The EPR measurement showed an initial increase in ROS formation in the cell by TQ. Thereafter, TQ interacts with mitochondria leading to a mild depolarisation without effect on ATP production. The overall oxidative stress level in the cell seems to stay unaltered. While the total ROS level is likely unchanged, the amount of reduced glutathione in the cell is significantly elevated by the treatment with TQ. Therefore, the antioxidative abilities of TQ described by other authors might be a result of the extended pool of reduced glutathione and not by TQ itself.

Since neuroblastoma cells seem to undergo the same adaptation, their TQ-induced cell degeneration may be attributed to the tremendously increased caspase-3 activity, which was observed. On the contrary, the lowered caspase-3 activity combined with the increased pool of reduced glutathione might be the mechanism for the enhanced resistance against oxidative stress in primary cell culture.

Taken together, the effects of TQ in cells stressed by ROS are beneficial, but does not appear to be mediated via restoring mitochondrial activity, and especially not via the radical scavenging properties of TQ.

## Data Availability

The data presented in this study are available in the article.
